# Polystyrene Nanocomposites Reinforced with Novel Dumbbell-Shaped Phenyl-POSSs: Synthesis and Thermal Characterization

**DOI:** 10.3390/polym11091475

**Published:** 2019-09-09

**Authors:** Lorenzo Abate, Francesco Agatino Bottino, Gianluca Cicala, Maria Assunta Chiacchio, Giulia Ognibene, Ignazio Blanco

**Affiliations:** 1 Department of Civil Engineering and Architecture and INSTM UdR, University of Catania, V.le A. Doria 6, 95125 Catania, Italy; 2 Department of Chemical Science, University of Catania, V.le A. Doria 6, 95125 Catania, Italy; 3 Department of Pharmaceutical Sciences University of Catania, V.le A. Doria 6, 95125 Catania, Italy

**Keywords:** polyhedral oligomeric silsesquioxanes, nanocomposites, POSS, polystyrene, thermal behavior, SEM analysis

## Abstract

Two series of novel dumbbell-shaped polyhedral oligomeric silsesquioxanes (POSSs), fully functionalized with phenyl groups at the corner of the silicon cages, were used to prepare polystyrene (PS) nanocomposites through the method of in situ polymerization. The percentage of the molecular filler reinforcement was set as 5% *w*/*w* of POSS and was checked by ^1^H-NMR spectroscopy. The obtained nanocomposites were characterized by thermogravimetric analysis (TGA), differential scanning calorimetry (DSC), Fourier transform infrared spectroscopy (FTIR) and scanning electron microscopy (SEM). Thermal and morphological properties were evaluated and compared among the nanocomposites obtained using the two different series of dumbbell-shaped POSSs and with the net PS. The thermal parameters for the prepared nanocomposites were very high when compared with those of neat PS, and they evidenced significant differences when an aliphatic or aromatic bridge was used to link the silicon cages. SEM analysis results allow us to hypothesize a justification for the different resistance to thermal degradation showed by the two series of molecular reinforcement.

## 1. Introduction

Since the middle of the last century, from common household goods to the important biomedical devices, by way of food packaging or building materials, polymers have made our lives easier and safer [1,2,3,4,5]. More recently, both for improving their properties and for reducing their environmental impact, eco-friendly inorganic materials have been added to polymeric matrices, thus leading to the production and commercialization of new polymer-based hybrid systems [6,7,8]. Literature has evidenced that property improvements cannot be obtained by a simple addition of an inorganic filler to the matrix [9], but a nanometric dispersion of the particles [10,11,12] and a good compatibility, obtainable via filler particle functionalization [13,14] are necessary. In the last few years, following these suggestions and by looking at the use of particular molecules like polyhedral oligomeric silsesquioxanes (POSSs) [15,16,17], our research group has been engaged in the design, synthesis and utilization of polymers reinforcement (polystyrene in particular) of POSSs [18,19,20]. This particular filler presents a hybrid nature due to its inorganic cage of silicon and oxygen atoms linked to organic groups by covalent bonds, thus making them molecules with the resulting advantages [21]. This aspect allows researchers to best use their nano-sized cage structures, dimensionally comparable to the most common polymer segments, thus producing an innovative chemical composition [22,23,24,25]. Starting from these considerations, we studied in the past several families of POSSs containing isobutyl, cyclopentyl and phenyl groups, with the aim of investigating whether and to what extent the physical properties of POSSs were modified by the introduction of these groups on the silicon cage [20,26,27]. We found that for phenyl-substituted POSSs, a higher resistance to the thermal degradation than cyclopentyl derivatives, and, for these last ones, a higher thermal stability than isobutyl-substituted POSSs. However, it has been well known in literature that the presence of phenyl groups increases the thermal stability of silicon cages [28,29], but their presence in a matrix decreases their compatibility, in comparison with aliphatic groups [30]. Hence, we had the need to verify how our POSSs, differently functionalized, behaved in a polymeric matrix—in particular in the polystyrene (PS) selected by us for this purpose. This need, combined with the aspiration to synthesize innovative POSS systems, which the literature has dealt with in the meantime, called “bridged” or “dumbbell-shaped” POSSs [31,32,33], induced us to verify the compatibility of this typology of hyper functionalized POSSs with a polystyrene matrix. Dumbbell-shaped POSSs aroused our interest because their change of organic bridge leads to a change of geometry, rigidity and symmetry of the obtained POSS, thus affecting their ability to be dispersed at a nanometric level [12,13,21].

In the present work, we synthesized the PS-based nanocomposites which had two different series of novel dumbbell-shaped POSS molecules (as fillers), fully phenyl functionalized in the periphery of the cage but linked with different aliphatic and aromatic bridges. The prepared PS/POSS nanocomposites were subjected to ^1^H NMR to check if the filler content fit with the desired one, whilst thermal stability was evaluated by means of thermogravimetric analysis (TGA) and differential scanning calorimetry (DSC). Finally, scanning electron microscopy (SEM) was performed to gain information about the POSSs dispersion within the polystyrene (PS) matrix, whilst Fourier transform infrared spectroscopy (FTIR) was used to analyze the residue obtained from the TGA experiments.

## 2. Experimental

### 2.1. Materials and Methods

1,2-bis(triethoxysilyl)ethane and 4,4′-bis(triethoxysilyl)biphenyl have been acquired from Aldrich Co. (St. Gallen, Switzerland) and used without further purification. A Na-benzophenone mixture was employed to distill the tetrahydrofuran (THF). 1,6-bis(triethoxysilyl)hexane, 1,10-bis(triethoxysilyl)decane and 4,4′-bis(triethoxysilyl)phenyl ether were obtained by using an excess of tetraethoxysilane (TEOS) in dry THF and the Grignard reagent according to the description reported elsewhere [34]. Trisilanolphenyl POSS was acquired from Hybrid Plastics Co. (Hattiesburg, MS, USA) and used without further purification. Styrene, 2,2-azobis (isobutyronitrile) (AIBN), methanol and toluene were acquired from Aldrich Co. (St. Gallen, Switzerland) and purified in our laboratory: Styrene was purified in an inhibitor removal column; AIBN was re-crystallised twice from dry ethanol at temperatures less than 40 °C and in a dark light condition; toluene was stirred over calcium hydride for 24 h and distilled in a nitrogen flow. 

Dumbbell-shaped POSSs, (Table 1 and Table 2), were synthesized according with the procedure reported elsewhere [35]. A 5% *w*/*w* POSS/styrene mixture, in toluene, was used for in situ free-radical polymerization: After being dissolved in toluene, AIBN was added to the POSS/styrene mixture, and then we proceeded to freeze everything using liquid nitrogen; after degassing by using a vacuum pump, we thawed it. This procedure was repeated three times, and thus the tube was sealed under vacuum and heated at 70 °C for 24 h under stirring. Finally, we obtained a solution that was poured in methanol, from which we collected, by filtration, the PS/POSS nanocomposite. The samples were then dried at 40 °C, under vacuum, obtaining yields of 82%, 83%, 88%, 86% and 79% for samples **1**, **2**, **3**, **4** and **5**, respectively. PS, used for comparison, was prepared by using the same procedure. 

### 2.2. ^1^H NMR Spectroscopy

Varian Unity INOVA equipment (^1^H 500 MHz, Varian, Palo Alto, CA, USA) was employed to check the exact filler content. Measurements were performed by using CDCl_3_ as a solvent and tetramethylsilane (TMS) as an internal standard. The evaluation was carried out by taking into account the ratio in the ^1^H NMR spectra among the POSS aromatic hydrogens and the polystyrene ones. 

### 2.3. Scanning Electron Microscopy (SEM)

**Figure 1:**
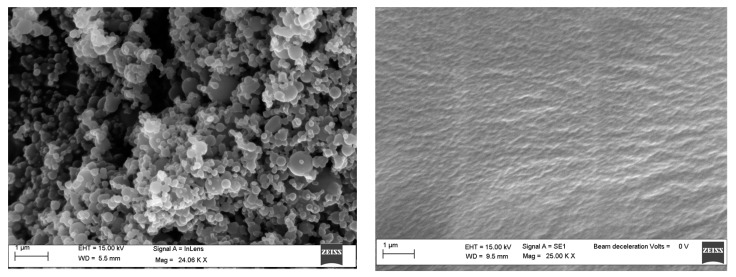
SEM images of polystyrene before (**left**) and after (**right**) drying in a stove.

The nanocomposites morphology was studied by SEM (ZEISS EVO MA 15, EVO-ZEISS, Cambridge, UK). In order to perform the analysis, an accelerating voltage of 15,000 KV was used. All the samples were treated overnight in an oven at 120 °C to remove any traces of toluene (Figure 1). Scans were carried out at different magnifications ranging from 500× to 25,000×.

### 2.4. Fourier Transform Infrared Spectroscopy (FTIR)

A Perkin Elmer Spectrum 100 spectrometer was employed in the determination of the FTIR spectra of the solid residues obtained from the TGA experiments at 700 °C. Spectra were carried out, without treatments, directly on samples at room temperature. For this purpose, we used an attenuated total reflectance (ATR) sampling accessory. Measurements were performed in the range from 4000 to 650 cm^−1^, with a resolution of 4.0 cm^−1^.

### 2.5. Differential Scanning Calorimetry (DSC)

Calorimetric investigations were performed, by using a Mettler DSC 1 Star System (Greifensee, Swizterland), for the determination of the glass transition temperatures (*T*_g_) of PS and PS/POSSs nanocomposites. The calibration of the DSC apparatus was carried out following a consolidated procedure [36], showing a very good agreement with the literature values of indium and tin [36,37]. The DSC scans were carried out from room temperature up to 200 °C, at a heating rate of 10 °C·min^−1^, on samples of about 6.0 × 10^−3^ g, held in sealed aluminum crucibles.

### 2.6. Thermogravimetric Analysis (TGA)

Thermal degradations were carried out in a Mettler Thermogravimetric Analyzer TGA 1 Star System (Mettler, Greifensee, Swizterland). For the equipment calibration, we considered the magnetic properties change of three metal standards (Isatherm, Nickel-alloy and Trafoperm 86, Mettler, Greifensee, Swizterland) at their Curie points (148, 355 and 750 °C), and we followed a consolidated procedure [38]. TGA scans were carried out by using 5 × 10^−3^ g of the sample at 10 °C·min^−1^ in the temperature range of 25–700 °C in a nitrogen (flow = 0.02 L·min^−1^) and a static air atmosphere. The International Confederation for Thermal Analysis and Calorimetry (ICTAC) Kinetics Committee recommendations were followed to correct the error in mass determination due to the reduction of the buoyancy (Archimedes) force with the temperature increase [39]. The percentage of the non-degraded sample, (1 − *D*)%, was reported as a function of the temperature, where *D* = (*W*_o_ − *W*)/*W*_o_, and *W*_o_ and *W* were the weights at the starting point and during the experiment, respectively. TGA scans were performed in triplicate, and the average value was considered.

## 3. Results and Discussion

After the in situ polymerization of styrene in the presence of 5% of POSS, the obtained composites were subjected to ^1^H NMR spectroscopy, with the aim to verify if the POSS percentage corresponded to the desired one. 

A difference between the actual POSS contents in the obtained samples and the theoretical content was observed, which could be explained by an oligomers formation in the reactant mixtures [40]. These oligomers, which are soluble in methanol, remained in the solution, thus resulting in a richer POSS. This occurrence justified the yields obtained and the POSS percentage in nanocomposites that are reported in Table 3 together with the thermal parameters.

Thermogravimetric (TG) experiments on PS and prepared nanocomposites were performed in dynamic heating conditions in the temperature range of 25–700 °C by using a scanning rate of 10 °C min^-1^, selected among the values normally used in this typology of experiments. 

**Figure 2:**
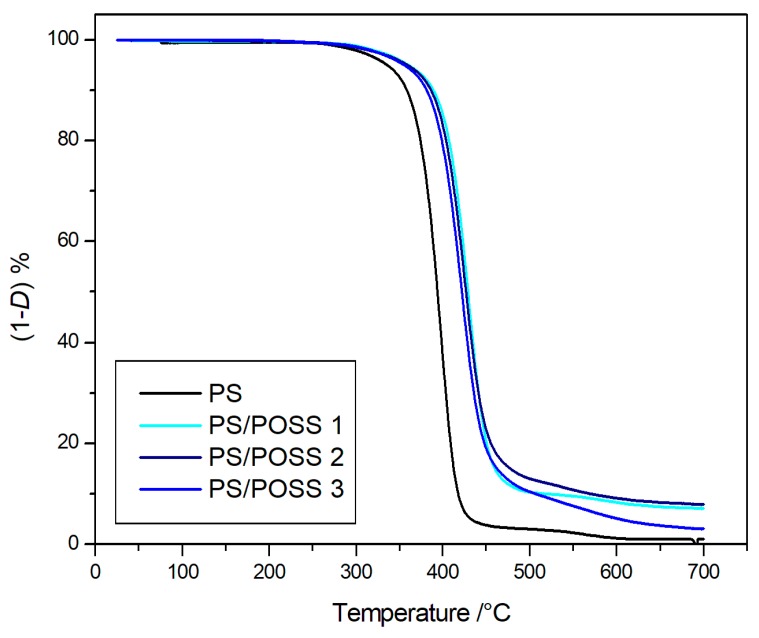
Thermogravimetric curves, in an inert atmosphere, for PS and samples **1**, **2** and **3**.

**Figure 3:**
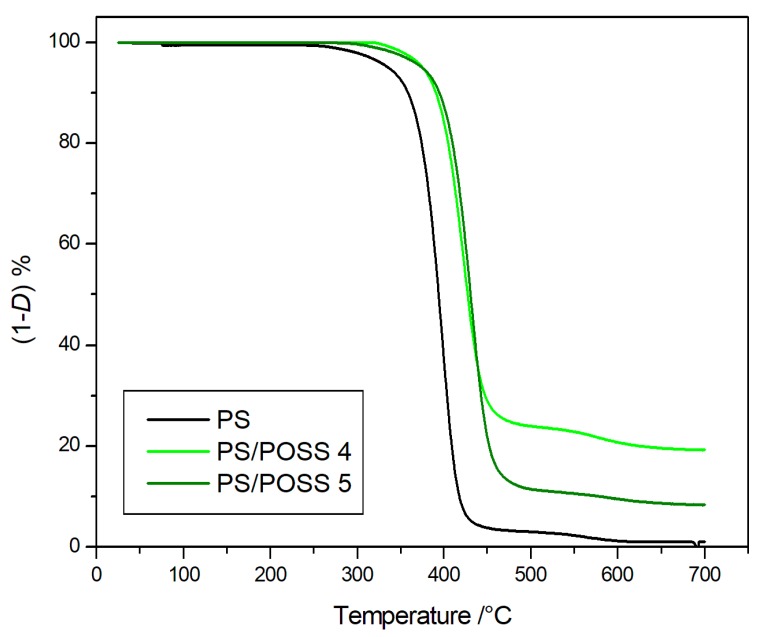
Thermogravimetric curves, in an inert atmosphere, for PS and samples **4** and **5**.

**Figure 4:**
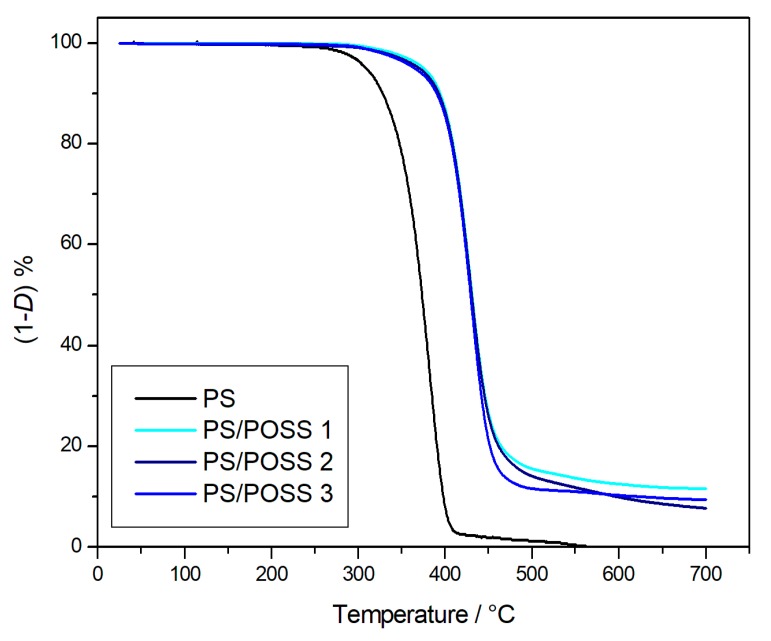
Thermogravimetric curves, in an oxidative atmosphere, for PS and samples **1**, **2** and **3**.

**Figure 5:**
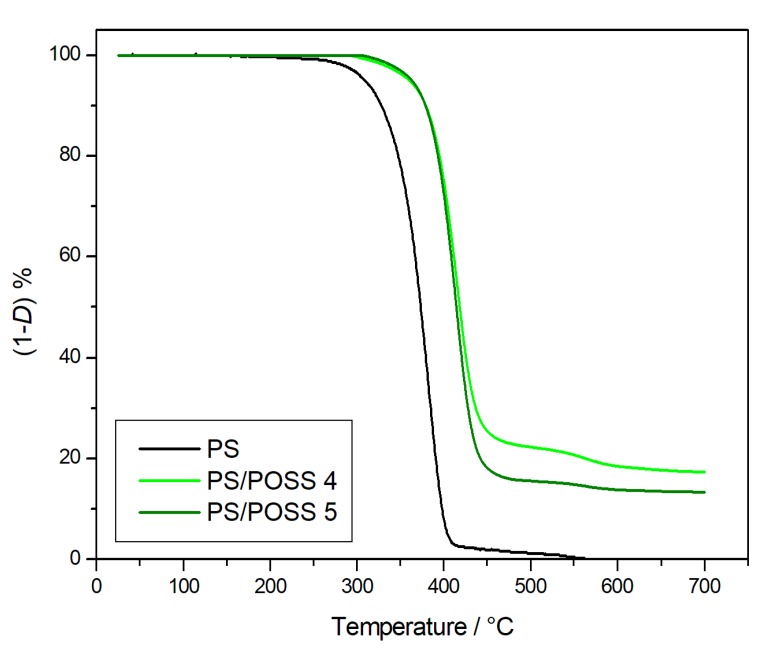
Thermogravimetric curves, in an oxidative atmosphere, for PS and samples **4** and **5**.

The degradations in inert environments were first performed, and the corresponding TG curves are reported in Figure 2 and Figure 3, whilst the TG curves obtained in oxidative conditions are reported in Figure 4 and Figure 5. Two parameters were considered for the assessment of the resistance to the thermal degradation, namely the temperature at 5% mass loss (*T*_5%_) and the solid residue at 700 °C. 

Both in the inert atmosphere and in the oxidizing one, the net PS reached complete degradation more quickly in air than in nitrogen, whilst all the nanocomposites evidenced the formation of a stable residue up to 700 °C (Figure 2, Figure 3, Figure 4 and Figure 5), ranging from about 10% for PS-reinforced with aliphatic bridged POSSs to about 20% for PS-reinforced by using phenyl bridged POSSs. The presence of a residue at 700 °C, a temperature at which PS was completely degraded in a quantity higher than the percentage of POSS in the matrix (Table 3) allowed us to speculate about a good dispersion of the POSS molecules in the matrix that resulted in an active interaction among the molecular filler and the PS. 

**Figure 6:**
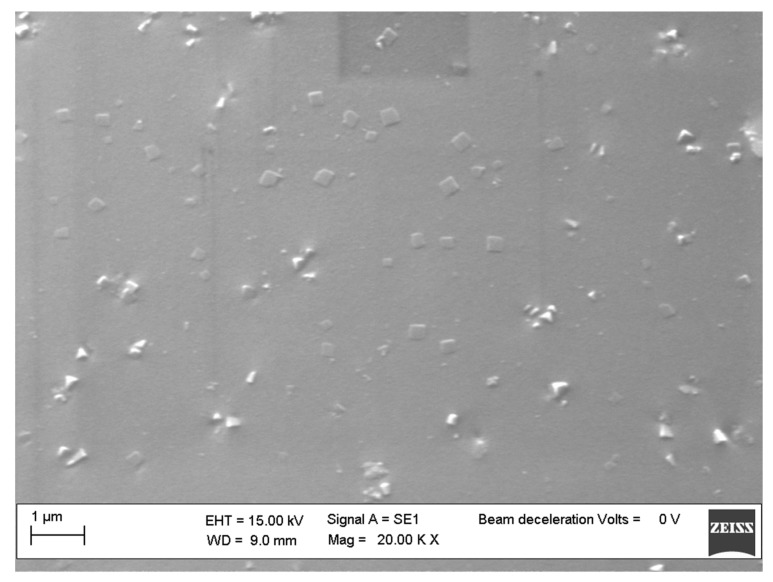
SEM images of PS/POSS **2** nanocomposites.

**Figure 7:**
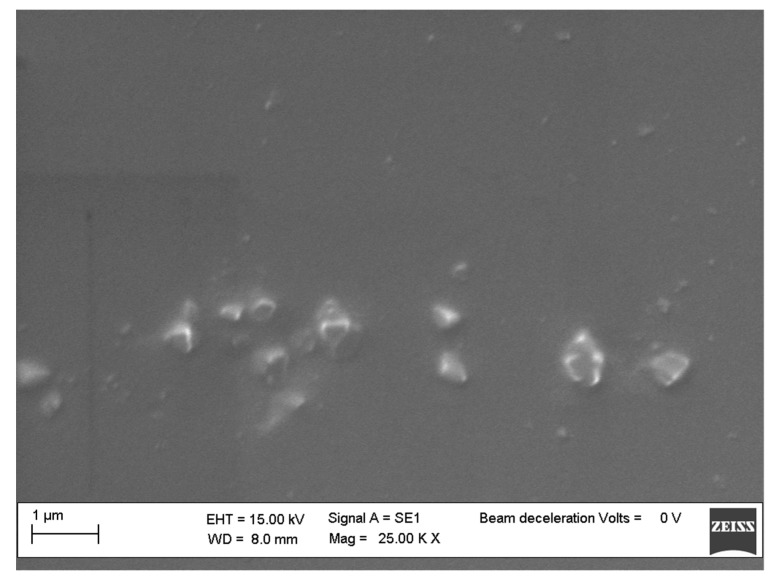
SEM images of PS/POSS **5** nanocomposites.

SEM investigation confirmed this hypothesis, showing a good dispersion of the functionalized silicon cages within the polymer (Figure 6). Probably due to the higher flexibility of the aliphatic bridges in respect to the aromatic ones that joint the POSS cages used to reinforce the polymer, a better dispersion of POSS **1**–**3** then for POSS **4** and **5** was observed within the matrix (Figure 7). 

POSS **4** and **5,** with their spatially blocked aromatic bridges, probably did not permit the same freedom of movement of the silicon cages in the dumbbell-shaped POSSs with aliphatic bridges, thus leading to a different symmetric/asymmetric structure that facilitated POSS auto-aggregation phenomena for samples **4** and **5** (Figure 7), which formed large block aggregates. 

The nanocomposites evidenced, both in nitrogen and in air, values of *T*_5%_ higher than those of the pristine polymer, Δ*T*_5%_ ≈ +35 °C in nitrogen and Δ*T*_5%_ ≈ +60 °C in air (Table 3). These high values of *T*_5%_, indicative of a good resistance to the thermal degradation, highlighted an increment of the thermal stability of the various nanocomposites in respect to the PS following the introduction of dumbbell-shaped POSSs in the matrix. 

**Figure 8:**
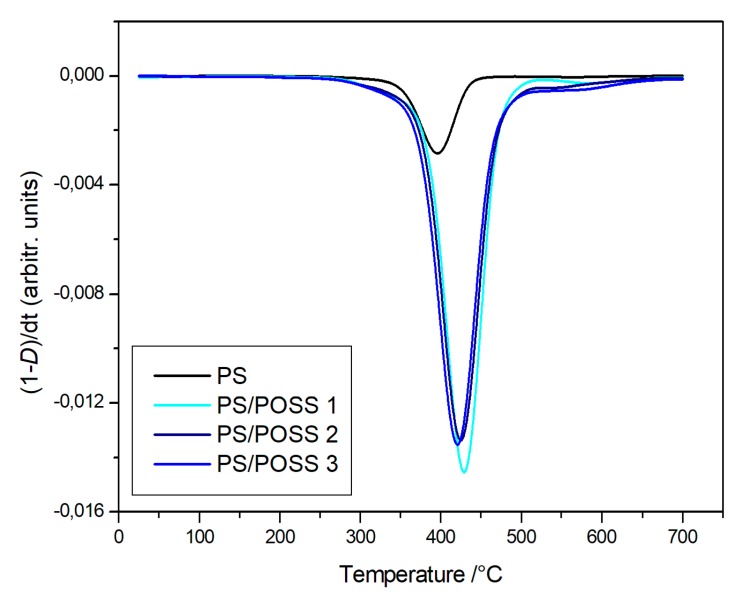
Differential thermogravimetric curves, in an inert atmosphere, for PS and samples **1**, **2** and **3**.

**Figure 9:**
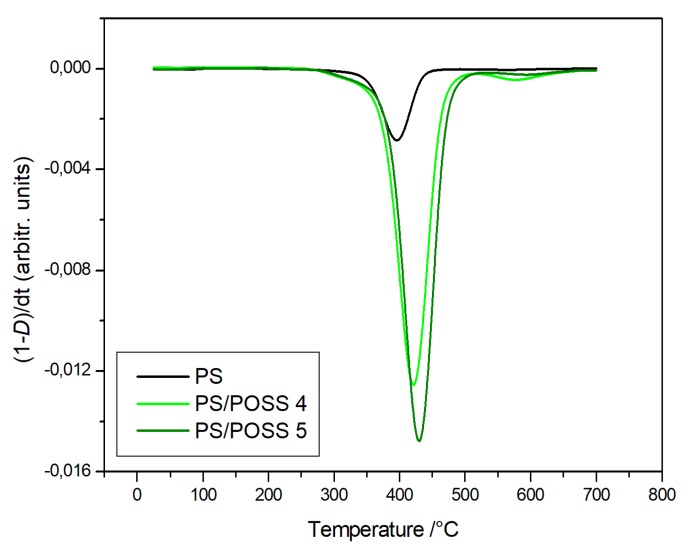
Differential thermogravimetric curves, in an inert atmosphere, for PS and samples **4** and **5**.

**Figure 10:**
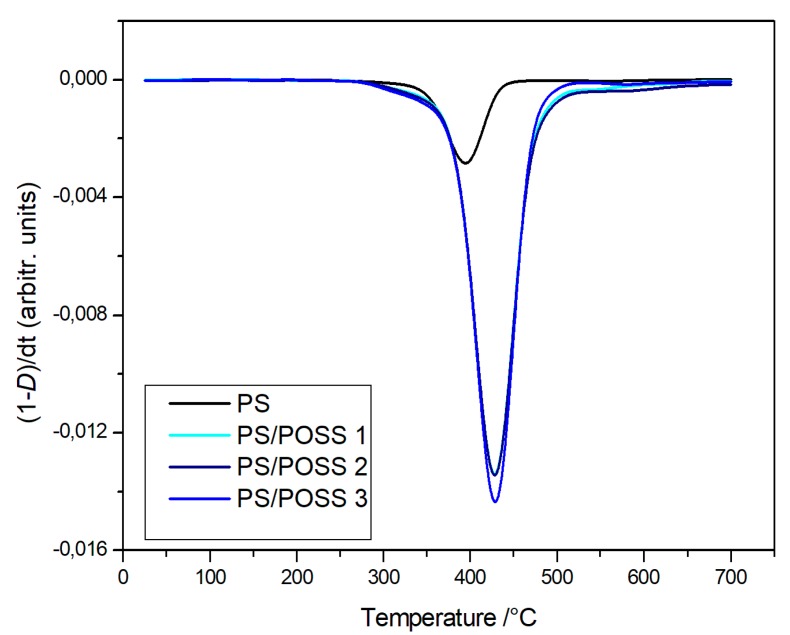
Differential thermogravimetric curves, in an inert atmosphere, for PS and samples **1**, **2** and **3**.

**Figure 11:**
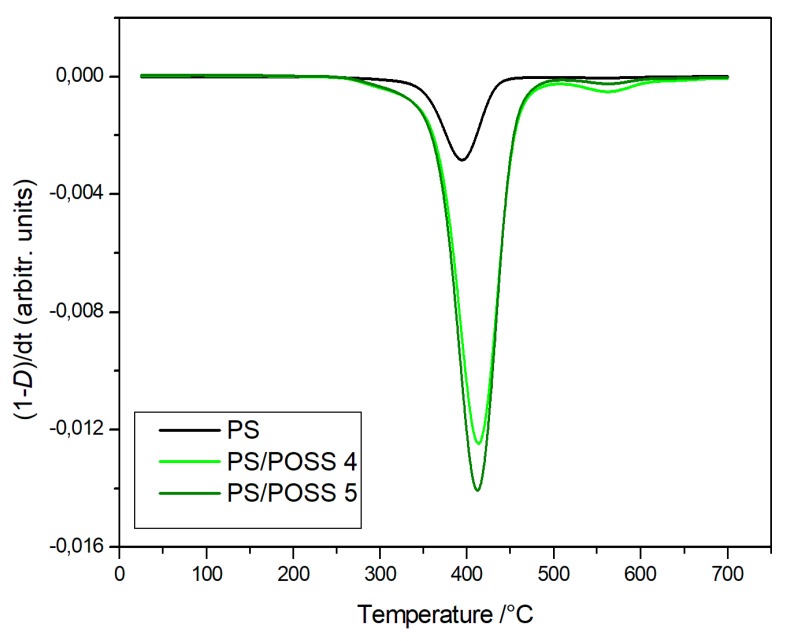
Differential thermogravimetric curves, in an inert atmosphere, for PS and samples **4** and **5**.

The degradations in oxidative and in inert environments proceeded similarly—both in the shape of TG curves and in the values at a temperature of 5% mass loss—that were comparable each other, meaning that the different atmosphere did not affect the degradation mechanism. Derivative thermogravimetric (DTG) curves are reported in Figure 8, Figure 9, Figure 10 and Figure 11 and show a main degradation step along with a second, smaller one at higher temperatures (550–600 °C) that was more evident for the nanocomposites reinforced with the phenyl bridged POSS, thus leading us to think that this stage may be due to POSS degradation. 

In order to gain information on the nature of the solid residue obtained at the end of TGA scans in both used atmospheres, we performed FTIR analysis, which evidenced only the presence of the silica characteristics peaks at about 1100 cm^−1^ (Figures S1 and S2).

**Figure 12:**
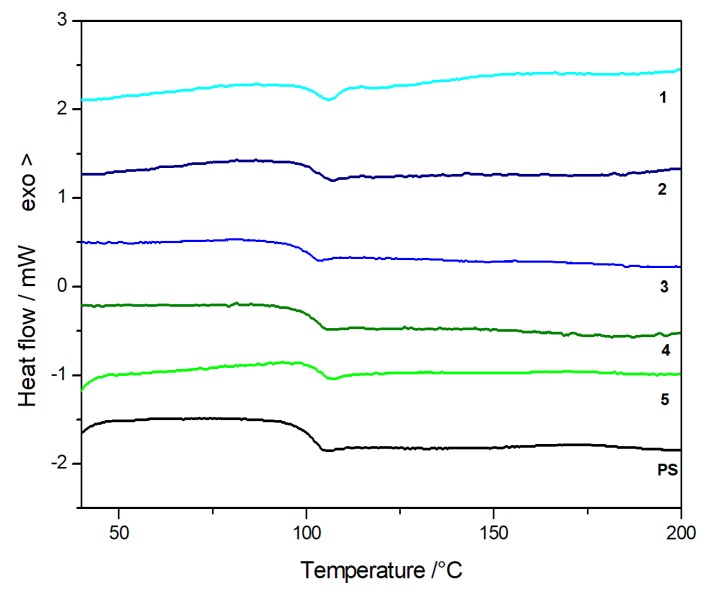
Differential scanning calorimetry curves for PS and PS/POSS nanocomposites.

Finally, the glass transition temperature was calorimetrically determined to better characterize the obtained compounds. DSC curves are reported in Figure 12, and the *T*_g_ values are reported in Table 3, showing a very small increment of the glass transition temperature for the nanocomposites in respect to virgin PS. This behavior could be attributed, in our hypothesis, to the nature of the groups at the POSS cage vertex. On considering that the incorporation of the POSSs on the polymeric chains change the matrix topology and alter its dynamics [41], the use of a less reactive phenyl-substituted POSSs during in situ polymerization may have had a mild effect on the reaction rate, thus not greatly altering the degree of crosslinking [42] and keeping the *T*_g_ values of composites quite constant in respect to that of neat polymer. 

## 4. Conclusions

The novel dumbbell-shaped POSSs, easily synthesized by a well-established methodology, have been proven as a valid reinforcement for the polystyrene matrix, showing a considerable improvement of the resistance to the thermal degradation of the prepared nanocomposites. We have shown encouraging SEM images regarding a good dispersion of the POSSs within the matrix, which has not always been reported in the literature for POSSs with a completely phenyl functionalized cage′s periphery. Finally, the environment in which degradation was performed (inert or oxidative) seemed to not influence the degradation mechanism that proceeded with two different stages, the first relevant one at a lower temperature than the second little one; this was attributed to the POSS degradation.

## Figures and Tables

**Table 1:** (**1**), (**2**) and (**3**) dumbbell-shaped octaphenyl polyhedral oligomeric silsesquioxanes (POSSs) structures, with aliphatic bridge, used in polystyrene (PS) nanocomposites.

C_86_H_74_Si_16_O_24_ (**1**)	C_90_H_82_ Si_16_O_24_ (**2**)
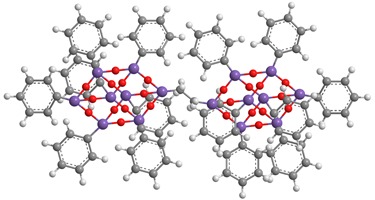	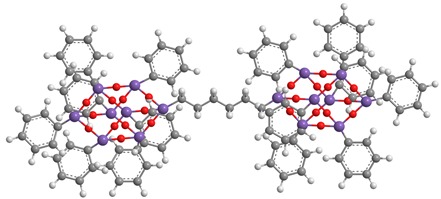
C_94_H_90_ Si_16_O_2_ (**3**)	
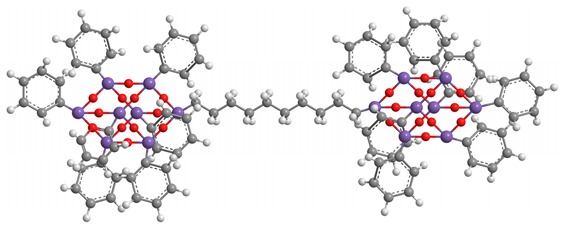	

**Table 2:** (**4**) and (**5**) dumbbell-shaped octaphenyl POSS structures, with aromatic bridge, used in PS nanocomposites.

C_96_H_78_ Si_16_O_25_ (**4**)
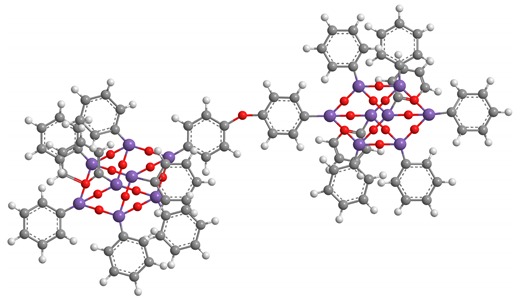
C_96_H_78_ Si_16_O_24_ (**5**)
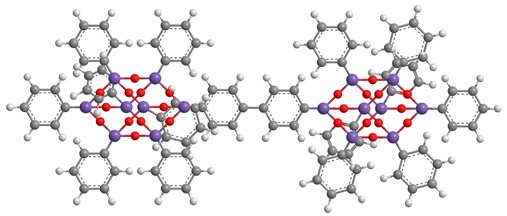

**Table 3:** POSS content (*w*/*w*), glass transition temperatures (*T*_g_), temperatures at 5% mass loss (*T*_5%_), residue percentage at 700 °C of PS, and nanocomposites in a static air atmosphere and in flowing nitrogen.

Compounds	POSS%	*T*_g%_ °C	Static Air		Nitrogen Flow	
			*T*_5%_ °C	Residue %	*T*_5%_ °C	Residue %
PS	/	101.2	309.0	0	341.3	0
**1**	5.96	102.6	375.7	11.6	360.0	7.1
**2**	5.82	103.5	370.3	7.8	359.3	7.9
**3**	5.12	101.7	364.7	9.4	355.3	3.1
**4**	6.01	103.5	363.2	13.3	374.8	10.4
**5**	5.60	102.5	360.3	17.3	375.7	19.3

## Supplementary Materials

The following are available online at https://www.mdpi.com/2073-4360/11/9/1475/s1.
