# Supplementary material for: Polystyrene Nanocomposites Reinforced with Novel Dumbbell-Shaped Phenyl-POSSs: Synthesis and Thermal Characterization
Source: Polymers (Basel). 2019 Sep 9;11(9):1475. doi: 10.3390/polym11091475 (PMC6780803; doi:10.3390/polym11091475)
Supplement: Supplementary file 1 [file polymers-11-01475-s001.pdf]

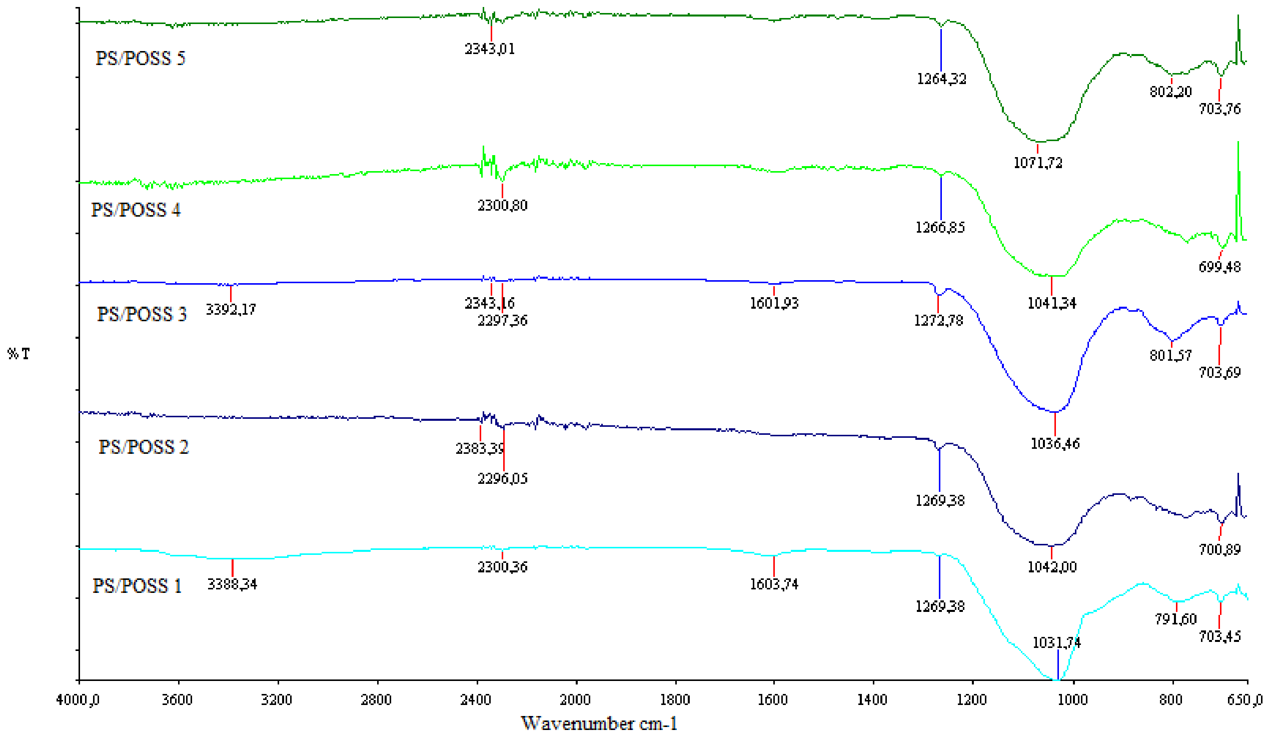

Figure S1. FTIR spectra of the residues at 700 °C, in flowing nitrogen, for the obtained nanocomposites.

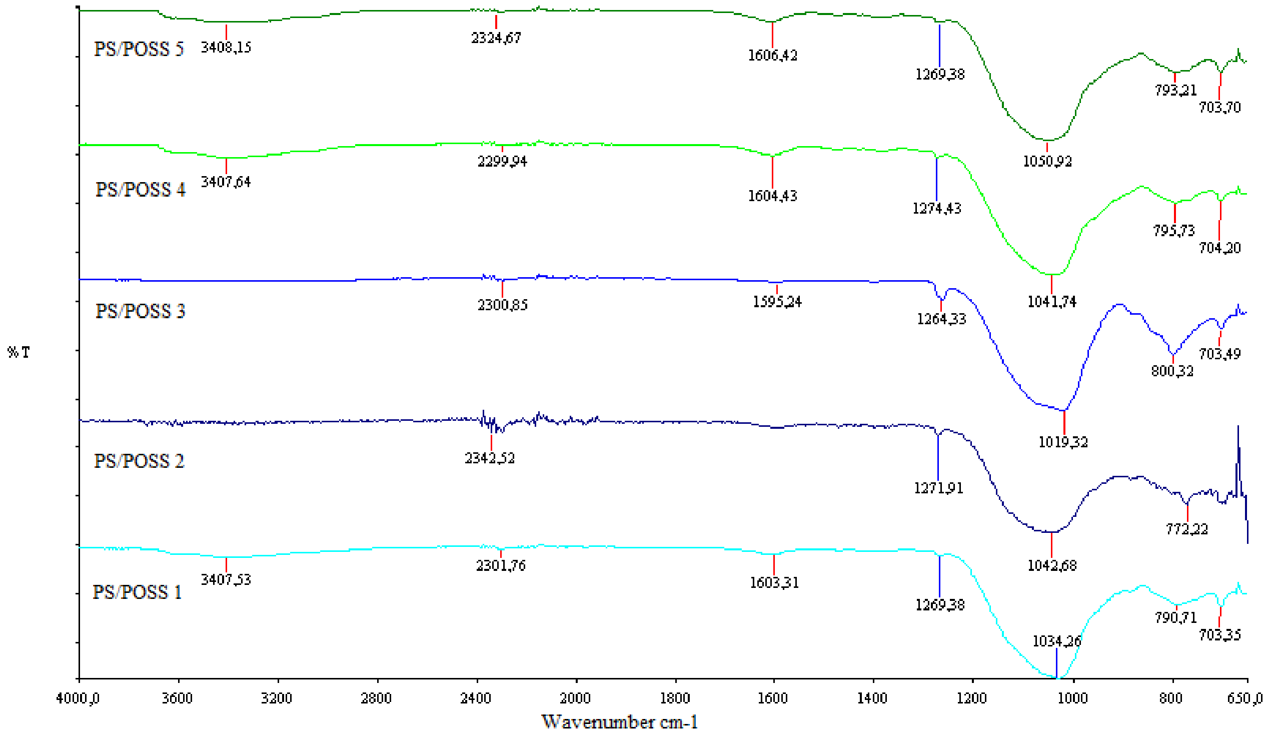

Figure S2 FTIR spectra of the residues at 700 °C, in static air atmosphere, for the obtained nanocomposites.
